# ApiAP2 Transcription Factors in Apicomplexan Parasites

**DOI:** 10.3390/pathogens8020047

**Published:** 2019-04-07

**Authors:** Myriam D. Jeninga, Jennifer E. Quinn, Michaela Petter

**Affiliations:** 1 Mikrobiologisches Institut—Klinische Mikrobiologie, Immunologie und Hygiene, Universitätsklinikum Erlangen, Friedrich-Alexander-Universität (FAU) Erlangen-Nürnberg, 91054 Erlangen, Germany; myriam.jeninga@uk-erlangen.de (M.D.J.); jennifer.quinn@uk-erlangen.de (J.E.Q.); 2 Department of Medicine, The University of Melbourne, Melbourne, VIC 3010, Australia

**Keywords:** Apicomplexa, *Plasmodium*, *Toxoplasma*, *Cryptosporidium*, malaria, gene regulation, transcription factor, ApiAP2, differentiation

## Abstract

Apicomplexan parasites are protozoan organisms that are characterised by complex life cycles and they include medically important species, such as the malaria parasite *Plasmodium* and the causative agents of toxoplasmosis (*Toxoplasma gondii*) and cryptosporidiosis (*Cryptosporidium* spp.). Apicomplexan parasites can infect one or more hosts, in which they differentiate into several morphologically and metabolically distinct life cycle stages. These developmental transitions rely on changes in gene expression. In the last few years, the important roles of different members of the ApiAP2 transcription factor family in regulating life cycle transitions and other aspects of parasite biology have become apparent. Here, we review recent progress in our understanding of the different members of the ApiAP2 transcription factor family in apicomplexan parasites.

## 1. Introduction

Apicomplexan parasites, like *Plasmodium* spp., *Toxoplasma gondii*, or *Cryptosporidium* spp. cause devastating diseases in humans and their eradication is still far away. *Plasmodium falciparum* parasites alone accounted for about 435,000 deaths in 2017 and small children under the age of five, in particular, are affected by the disease [1]. *T. gondii* causes many deaths in immunocompromised patients and it has detrimental effects when newly acquired during pregnancy [2], and *Cryptosporidium* spp. are one of the leading causes of infant diarrhoea in developing countries [3]. With emerging drug resistance in *Plasmodium* and other apicomplexan parasites, treatment options are becoming limited [4,5], and it is clear that there is a need for better and innovative drugs. This will be greatly aided by a profound understanding of the parasites’ biology. The apicomplexan parasites have complex life cycles, which are marked by several differentiation steps that are associated with significant switches in the transcriptome [6,7,8,9,10]. In *Plasmodium* and *Toxoplasma*, epigenetic regulation via histone modifications and histone variants has been implicated in the control of gene expression (reviewed in [11]), but also, generally, specific transcription factors (TFs) are of major importance in the developmental regulation of transcription in eukaryotes [12]. For a long time, only few conserved DNA binding domains of specific TFs were identified in the apicomplexan lineage, including myb (myeloblastosis), PREBP (Prx Regulatory Element binding protein), HMGB (high mobility group b), C2H2 zinc fingers, and E2F (only in *Cryptosporidium*) domains [13,14,15,16,17,18,19,20]. However, in 2005, Balaji et al. first described a class of putative TFs in Apicomplexa that carried a domain presenting similarity to the Apetala2/ERF (ethylene response factor) (AP2/ERF) integrase DNA binding domain, which is present in many plant TFs [21,22]. Thus, this novel family of putative TFs was dubbed ApiAP2 (apicomplexan AP2). The putative acquisition of an ancestral AP2/ERF TF from the plant lineage by horizontal gene transfer was in line with the hypothesis that a red alga of rhodophyte origin was an endosymbiont in the apicomplexan parasites ancestor, from which the apicoplast organelle originated [23,24,25,26].

*P. falciparum* codes for 27 putative ApiAP2 TFs, while in *T. gondii* and *C. parvum*, there are more than 60 and 10 ApiAP2 annotated genes, respectively [27]. Some of the ApiAP2 TFs are widely conserved among the apicomplexan families and their close relative *Perkinsus marinus* [28], suggesting that their common chromalveolate ancestor could already have had about 9 to 18 AP2 domain containing TFs, while the more species specific factors likely evolved through independent lineage-specific expansion after the divergence of the Apicomplexan species [21,28]. Gene expression data from *P. falciparum* and *C. parvum* indicated that different ApiAP2 family members were expressed in different stages during parasite development, suggesting that they might be involved in life cycle progression and differentiation processes, like their plant homologues [21,29]. Numerous studies have since corroborated this hypothesis and demonstrated the critical importance of ApiAP2 TFs in apicomplexan biology.

## 2. Structure of ApiAP2 Transcription Factors

**Figure 1:**
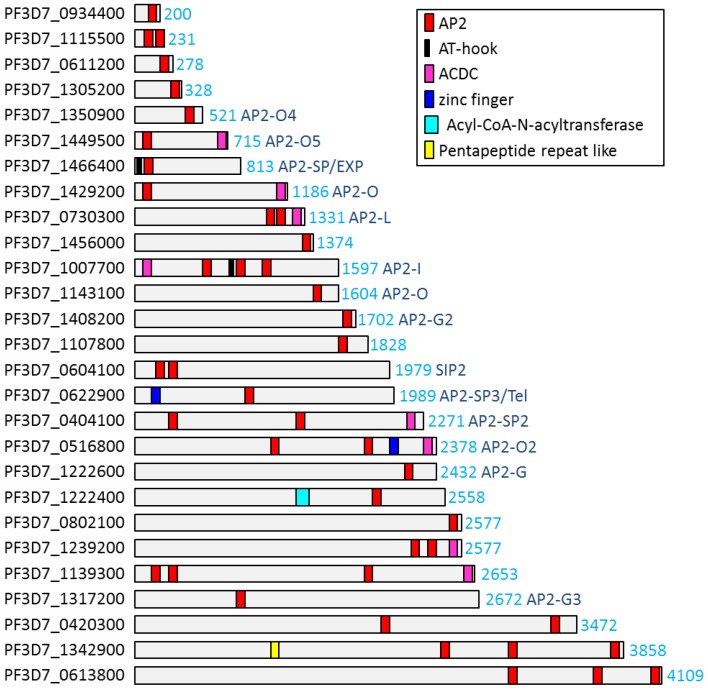
Apicomplexan AP2 (ApiAP2)-domain-containig proteins in *P. falciparum*. The proteins are organised by size, ApiAP2-domains are marked in red, ACDC (AP2-coincident domain mainly at the C-terminus) domains in pink, AT hooks in black, zinc finger in blue, Acyl-CoA-N-acyltransferase in light blue, pentapeptide-repeat-like domains in yellow.

The ApiAP2 TFs can comprise one to four AP2 domains as well as additional functional regions and they greatly vary in size, from approximately 200 to several thousand amino acids (Figure 1). There is generally little sequence homology outside of the AP2 domains. Apart from the AP2 domain, other protein domains that are present in some of the ApiAP2s in *Plasmodium* spp. include a DNA binding domain called AT-hook, a zinc finger domain, an Acyl-CoA-N-acetyltransferase domain, as well as a pentapeptide-repeat-like domain (Figure 1). Several ApiAP2 TFs in *P. falciparum* additionally contain a conserved motif that was named the ACDC domain (AP2-coincident domain mainly at the C-terminus), but the function of this domain is unknown thus far [30].

AP2 domains are about 60 amino acids in size and they normally consist of 3 β-strands and one C-terminal α-helix that stabilises the β-strands [21,31]. An insert is present between strand 2 and 3 that might stand out as a hairpin-like structure, which is positively charged. This positive charge probably enables the nonspecific interaction with the DNA backbone, enhancing the affinity of the ApiAP2 TF to the DNA. When comparing multiple AP2 domains from plants and other organisms, it was shown that 12 amino acid residues were highly conserved, which are implicated in the formation of specific DNA contacts with GCC boxes and DNA backbone contact [21]. In plants, the residues corresponding to R150, R152, W154, E160, R162, R170, and W172 (in the AP2 domain of the plant TF ATERF) mediate the specific binding to the GCC boxes. In ApiAP2s, the R150 residue is often changed to be Y or S and the R152 residue, rather, is found to be D or N, with both being more likely to interact with the amino group of adenine than with guanine. In contrast, the E160 and W172 residues are maintained as polar amino acids with aromatic residues, indicating that they are less important for differential sequence specificities. Altogether, this shows that there is a higher variability of binding and recognition motifs for the ApiAP2 TFs, which may represent specific adaptation to the higher AT content of the apicomplexan parasites [21]. The crystal structure of PfAP2-Sp/Exp (PF3D7_1466400) in complex with DNA revealed binding of the double stranded DNA in the major groove of the ApiAP2 factor and the dimerisation of two ApiAP2 domains by domain-swapping of the α-helices, so that the α-helix from one ApiAP2 aligns with the β-strands of the other ApiAP2 [31]. This dimerization is probably induced by DNA binding and it is stabilized by disulphide bridges that are formed at the cysteine residue C76, with a nearby proline residue further facilitating it. Several other residues are also important for DNA binding; among these were the N72, R74, R88, and S90 [31]. Furthermore, the AT-hook contributes to DNA binding but in a non-sequence-specific way [31,32].

## 3. DNA Binding Motifs of ApiAP2 Transcription Factors

The regulation of gene expression by specific TFs is generally mediated by the interaction of the DNA binding domain with specific DNA motifs that are present in regulatory regions upstream of the target gene promoters. In *P. falciparum*, the motifs that were bound by 24 AP2 domains in 20 ApiAP2 TFs were revealed by protein binding microarrays [33,34], and several of these motifs have been functionally confirmed in subsequent studies [35,36,37,38]. These ApiAP2 binding motifs are largely present in intergenic regions that are upstream of genes, and often more than one motif can be found clustered together, indicating the combinatorial regulation by ApiAP2 TFs. Several AP2 domains bind similar motifs, such as the CACACA repeat [38], but the different AP2 domains that are present in single ApiAP2 TFs generally recognise distinct motifs, which could help in restricting the specificity of the target gene repertoire, despite redundancy in sequence recognition [33]. In line with the hypothesis that ApiAP2s mediate developmental progression, some of these motifs were enriched in genes that showed similar expression profiles across the asexual replication cycle and might thus represent co-regulated target genes of specific ApiAP2s. A study that addressed the accessibility of DNA motifs during the life cycle using a Tn5 transposase mediated approach (ATACseq) further supported this concept by identifying sequences that correlated with stage specific gene expression and that were bound by ApiAP2 TFs [38]. Another study suggested that the accessibility of these motifs correlates with certain histone modification patterns, linking ApiAP2 factors to epigenetic gene regulation [39].

Similar to other Apicomplexa, *Cryptosporidia* show highly regulated gene expression patterns throughout their intracellular life cycle. Oberstaller et al., 2013 predicted putative TF binding motifs through the identification of overrepresented motifs in the upstream region of genes with similar expression patterns [29,34]. The E2F-like motif TGGCGCCA was among these, which could be utilised by the E2F TFs that are present in *Cryptosporidium*, but are absent in *Plasmodium* spp. and other Apicomplexa. Other motifs included the G-box motif GxGGGG, which was shown to be recognised by cgd8_810 using protein binding microarrays [29]; the (T/C)GCATGC(A/G) motif that is bound by cgd2_3490 and was also characterised as binding motif for the PfAP2-EXP and its rodent orthologue PbAP2-SP [34]; and the uncharacterised motif (A/C)AACTA [29]. Protein binding microarrays further showed that the TGCAT motif (also dubbed AP2_1-like) present in the binding site of cgd2_3490 was also recognised by three other ApiAP2-domain-containing proteins. Showing similar binding redundancy, the CACACA motif was recognised by four different ApiAP2 TFs and the G box motif was recognised by three different proteins. Interestingly, cgd1_3520 recognised both, the AP2_1-like and the G-box motif [28]. This suggests a high redundancy of ApiAP2 factors in *Cryptosporidium* that may possibly be balanced by the usage of specific E2F TFs in comparison to other Apicomplexa [28].

## 4. Developmental Transitions during Apicomplexan Life Cycles

**Figure 2:**
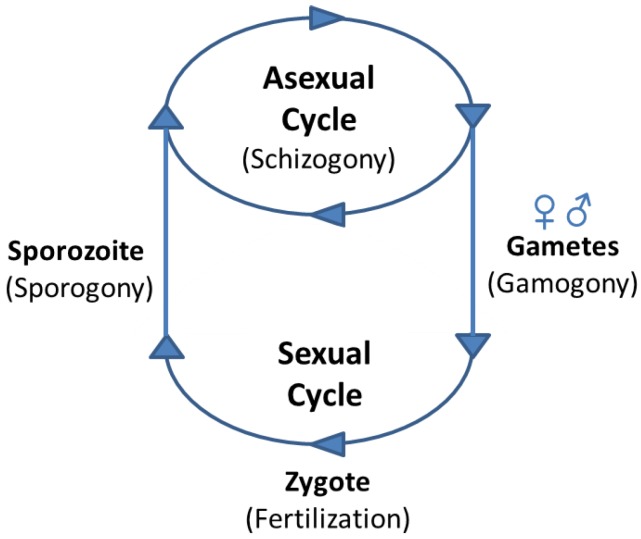
Simplified schematic life cycle depicting major developmental transitions during apicomplexan parasite development, which involve regulation by ApiAP2 transcription factors (TFs).

The life cycles of Apicomplexan parasites are characterised by several common major differentiation steps, during which the parasites transition into morphologically and physiologically very distinct forms: first from sporozoites, which facilitate the transmission to a new host, to asexual forms that undergo cycles of replication by schizogony. Some of the progeny differentiate to the sexual forms, male and female gametes, which then fertilize and give rise to the zygote that develops into an oocyst, in which sporogony occurs (Figure 2). Significantly different gene expression profiles characterize all these different stages [10,40,41,42,43,44,45,46,47,48,49,50,51,52,53,54], which will be described in detail for each parasite genus in the following paragraphs.

Several species of Anopheles mosquitos transmit *Plasmodium* parasites. During the bite of an infected mosquito, sporozoites are injected from the salivary gland into the human bloodstream, from where they invade hepatocytes (reviewed in [55]). There, the sporozoites develop into the liver schizont stage and propagate until 10,000–30,000 newly formed merozoites egress into the bloodstream. This hepatic cycle is then followed by the asexual intraerythrocytic cycle, in which the released merozoites invade erythrocytes, where they develop from a ring stage form into a trophozoite that undergoes schizogony. Finally, 16–32 new merozoites egress from the mature schizont and infect new cells, resulting in the asexual replication cycle, which is responsible for the clinical symptoms of malaria. For transmission to a new host via a mosquito, the parasite needs to undergo sexual differentiation into male or female gametocytes. The development of the gametocytes encompasses five morphologically distinct stages and takes approximately 12 days in *P. falciparum* [56]. In contrast, gametocytes of the rodent malaria parasites *P. berghei* and *P. yoelii* only differentiate in 48 h. It is presumed that all gametocytes that originate from one schizont develop into the same sex [57]. A mosquito can finally ingest the mature gametocytes during a blood meal. Conditions in the mosquito midgut trigger gametogenesis and male and female gametes egress from the blood cells (reviewed in [58]). During this process, the male gametocytes rapidly divide into eight microgametes that exflagellate and then fertilise the female macrogametes, forming a motile zygote called ookinete. The ookinete undergoes meiosis and it is the only polyploid phase of the parasite life cycle [59]. The ookinete then exits through the peritrophic membrane and migrates across the midgut wall to settle on the other side as an oocyst (reviewed in [60]). Inside the oocyst, the parasite again asexually propagates until a generation of new sporozoites is released, which migrate to the mosquito’s salivary glands to be secreted with the next bite.

The heteroxenous parasite *T. gondii* is mainly transmitted by the ingestion of contaminated food or water. Humans can be either infected by ingestion of tissue cysts that are present in undercooked meat, or by faecal/oral transmission of sporulated oocysts shed by the feline definite host. Diaplacental transmission also occurs, which is why first time infection during pregnancy can have detrimental effects on the unborn baby. The sexual cycle of *T. gondii* takes place in the intestine of a feline definitive host, where the bradyzoites that are released from ingested tissue cysts invade gut epithelial cells and then undergo schizogony and gametogenesis. Fertilization results in the formation of a sporulated oocyst, which is shed in the faeces and can be taken up by an intermediate host (including humans), in which the asexual cycle takes place. There, the sporozoites that are released from the oocysts differentiate into fast replicating asexual cells, called tachyzoites, which can invade almost any nucleated cell, leading to their dissemination into other tissues, such as brain, muscles, or placenta. The parasites can then differentiate into nearly dormant and slowly replicating bradyzoites, which form a tissue cyst and represent a transmission stage, either back to the definitive host or another intermediate host [61].

*Cryptosporidia* have a similar life cycle to *Plasmodium* or *Toxoplasma*, however they are monoxenous, as transmission occurs via ingestion from the environment. In their life cycle, the asexual sporulated thick-walled oocyst is taken up and forms four sporozoites that invade gut epithelial cells [62], in which the asexual cycle takes place. Within the epithelial cells, the sporozoites undergo merogony to form type I meronts (within 24 h after infection) that egress as merozoites (about 36 h post-infection) and re-infect new gut epithelial cells. Subsequently, the merozoites undergo a second cycle of merogony to form either type I meronts again, or type II meronts, which undergo gametogenesis to form micro- and macrogametes [63]. These fertilise and form the zygote, which further develops either into the thick-walled oocyst that is shed into the environment or into the thin-walled oocyst that auto-infects the gut cells [62].

In the following paragraphs, we would like to review what is known regarding the regulation of the transcriptome by ApiAP2 TFs during these life cycle transitions of apicomplexan parasites.

## 5. ApiAP2s of Malaria Parasites

### 5.1. Asexual Stages

During the intra-erythrocytic developmental cycle (IDC) of *P. falciparum*, gene expression occurs in a regulated, cascading manner, so that each gene is only expressed during a restricted time frame [6]. This is also true for ApiAP2 TFs, which suggests that ApiAP2 expression during the IDC induces a regulatory cascade that results in sequential activation of ApiAP2 TFs. In support of this concept, binding motifs for many ApiAP2 TFs are present upstream of other ApiAP2 genes [33]. Systematic knock out studies in the rodent malaria parasites *P. berghei* and *P. yoelii* have consistently identified ten ApiAP2 TF members as being refractory to gene disruption, indicating that they might be essential during the IDC (Table 1) [64,65,66]. However, several targeted studies have recently shed light on the functions of selected ApiAP2 proteins during intra-erythrocytic growth.

AP2-I (PF3D7_1007700) encodes a 190 kDa protein with three AP2-domains, an AT-hook, and the ACDC domain. AP2-I is the only ApiAP2 TF in which the ACDC domain is located in the N-terminal region [33,82]. Five acetyl-lysine sites were found in AP2-I, of which two lay in the AP2 domains AP2-D1 (K555) and AP2-D2 (K804). The mutation of K804 to glutamine resulted in a loss of specific DNA binding by the AP2-D2, indicating that neutralization of the positive charge by acetylation may provide a regulatory mechanism to relieve DNA binding [82]. This was similar to previous reports on DNA-binding of TF in other organisms.

All three AP2 domains of AP2-I are associated with different DNA motifs (Table 1) [33]. The DNA binding motif of its AP2-D3, GTGCAC, is enriched upstream of many schizont expressed genes with putative functions in erythrocyte invasion [33,83,84,85], implicating AP2-I in invasion gene regulation. Indeed, chromatin immunoprecipitation, followed by deep sequencing (ChIPseq), confirmed that AP2-I associates with many invasion gene promoters [37]. This interaction was independent of the other two AP2 domains, as the mutation of critical residues in these domains had no effect on AP2-I binding or AP2-I target gene expression. Interestingly, AP2-I binds its own promoter, suggesting that it may enhance its own expression. Five other ApiAP2 TFs with expression peaks immediately after AP2-I during the IDC were also targets of AP2-I, as consistent with the regulation of developmental progression by an AP2 TF cascade [37]. In several studies, AP2-I was found in complex with acetyl-lysine binding bromodomain proteins (PfBDP1 and PfBDP2) and other chromatin remodelling factors [37,38,86]. Intriguingly, AP2-I binding precedes the recruitment of the bromodomain protein PfBDP1, which is also critical for invasion gene regulation [86]. Thus, AP2-I likely exerts its regulatory function by inducing chromatin remodelling and recruiting the transcriptional machinery; however, a complete understanding of the molecular details requires further investigations.

Another ApiAP2 TF that has been indirectly linked to the process of invasion is the 485 kDa PF3D7_0613800. PF3D7_0613800 expression peaks in the late trophozoite to schizont stage and it was identified by mass spectrometry as an interaction partner of PfBDP1 [37,86]. The pull down of all three proteins with a novel DNA motif that was identified by ATACseq as accessible to DNA binding proteins, further supported the connection between PF3D7_0613800, AP2-I, and PfBDP1 [38]. In the same study, Pf3D7_0613800 was pulled down as the only TF binding to the DNA motif GAGCTCAA. Interestingly, this motif is different from the binding sites that were predicted for two of its three AP2 domains [33], indicating that the third AP2 domain, for which no binding site was previously predicted, might recognize this motif. In an ApiAP2 KO screen, the *P. berghei* orthologue of PF3D7_0613800, PBANKA_0112100 was shown to be refractory to deletion, indicating that this TF might be essential for blood stage replication [65]. Moreover, PF3D7_0613800 was implicated in drug resistance, as mutations in amino acid Q197 of PF3D7_0613800 were associated with resistance to three different MMV (Medicines for Malaria Venture) compounds in a chemogenetic screen that aimed to identify novel drug resistance mechanisms [87]. Variants of this gene were also linked to quinine resistance in a genome-wide association study [88], providing further evidence for a potential role of Pf3D7_0613800 in the response to drugs. Further, PF3D7_0613800 was down-regulated upon re-entry into the cell cycle following DFMO (difluoromethylornithine) induced cell cycle arrest, indicating that it might also have a functional role during the G1 phase of the parasite cell cycle [89].

PfAP2-EXP (PF3D7_1466400) is a 92 kDa protein that contains a single AP2 domain and an AT-hook site at its N-terminal end [79]. The acetylation of lysine 11 has been reported, however the functional significance of this has not been established [79,82]. The DNA motif recognized by PfAP2-EXP, TGCATGCA is the most overrepresented octamer in intergenic regions in Apicomplexan genomes [90,91], including *P. falciparum*, *T. gondii*, *C. parvum* (where it is bound by cgd2_2490), and *Eimeria tenella* [34]. Binding sites of PfAP2-EXP have been predicted upstream of the *var* gene family members [33], but also upstream of most of the ApiAP2 genes [34]. While the complete disruption of PfAP2-EXP failed, truncation produced viable parasites that showed an upregulation of members of the clonally variant exported protein families PfMC-2TM, RIFIN, and STEVOR [79]. PfAP2-EXP shares 92% identity in the AP2 domain with its *P. berghei* orthologue PbAP2-Sp, which was shown to be dispensable for asexual growth and has a function in sporozoites (discussed later) [78]. Thus, this species-specific difference may rely on the more divergent C-terminus [79].

Unlike other ApiAP2 TFs, PfSIP2 (PF3D7_0604100) was implicated in heterochromatin formation and genome integrity rather than transcriptional regulation [36]. The 230 kDa protein contains two AP2-domains that are arranged in tandem at its N-terminus, and it is processed to an active 60 kDa N-terminal fragment during schizogony. The AP2-D1 of PfSIP2 is sufficient for binding to the SPE2 motif [33,34,36], which is located in tandem arrays approximately 2 kb upstream of the subtelomeric upsB-type *var* genes [36,92]. Orthologues of PfSIP2 can be found in all sequenced *Plasmodium* species, such as *P. berghei* (PBANKA_0102900), *P. yoelii* (PY17X_0104500), as well as *C. parvum* (cdg4_3820). However, subtelomeric SPE2 arrays are limited to *P. falciparum*, whereas in other species, SIP2 consensus sequences are mainly located within chromosome internal regions, indicating that SIP2 likely has additional functions to chromosome end biology. So far, SIP2 was refractory to deletion in *P. falciparum* and rodent parasites; thus, PfSIP2 could be essential in parasite survival in the IDC [36,64,65]. The overexpression of the N-terminal fragment of PfSIP2 had only marginal effects on gene expression, further corroborating that its essential function is unlinked to transcriptional regulation [36].

Another ApiAP2 family member implicated in telomere biology is the protein that is encoded by PF3D7_0622900, which was recently identified as a component of the *P. falciparum* telomere-binding protein complex and thus dubbed PfAP2-Tel [68]. PfAP2-Tel is expressed throughout the intra-erythrocytic developmental cycle (IDC), during which it co-localizes with telomeric clusters at the nuclear periphery, which is consistent with its specific binding to the telomeric repeat motif GGGTT(T/C)A on all 14 chromosomes of *P. falciparum* [68]. ApiAP2-Tel possesses an atypical AP2 domain with low sequence identity (<27%) to other members of the ApiAP2 family. This AP2 domain lacks one of the three β-sheet structures, which are considered to be essential for DNA-binding [31], but PfAP2-Tel binding to the telomeric repeat motif GGGTT(T/C)A was experimentally confirmed in electro-mobility shift assays (EMSA). Interestingly, PfAP2-Tel was pulled down together with PfBDP1 [86], providing another link between ApiAP2 TFs and chromatin modifications. The orthologue of ApiAP2-Tel was successfully disrupted in rodent malaria parasites, indicating that its function in asexual parasites is non-essential (see Section 5.3 for more details) [64,65,66].

### 5.2. Sexual Differentiation

To continue the life cycle and ensure the transmission of the parasite, a proportion of the fast-proliferating asexual cells differentiate into dimorphic sexual forms—the male and female gametocytes. Remarkably, the differentiation process in *P. falciparum* occurs in about 10 to 12 days, whereas it is much faster in other *Plasmodium* species. The commitment step, which precedes the differentiation, is associated with a transcriptional switch, which is mediated by the ApiAP2 TF AP2-G [50,72,73]. As the gametocytes can only propagate further inside the mosquito host, the conversion rate is tightly regulated and it depends on environmental factors to which the parasites can adapt. On the one hand, it was shown that the conversion rate in high transmission areas is lower than in low transmission areas, which is probably due to selective pressure towards the optimisation of transmission in these areas [93]. Additionally, commitment to sexual differentiation is a disadvantage in asexual growth, as becomes clear from high mutation rates of AP2-G in culture and from AP2-G knockout parasites, which outgrow their wildtype parents [65,72,94,95]. On the other hand, the conditions in the vertebrate host also influence the conversion rate; for instance, high concentrations of homocysteine, low levels of LysoPC (caused by high parasitemia), as well as other factors lead to higher differentiation rates [96,97].

In *P. falciparum*, PfAP2-G (PF3D7_1222600) was initially found by comparing the transcriptional variation in clones of two 3D7 stocks (3D7-A and 3D7-B) [98]. Until then, only a deletion in the subtelomeric region of chromosome 9 or defects in several early gametocyte genes had been associated with the disability to form gametocytes [74,99,100]. However, the region on chromosome 9 was present in the two non-gametocyte forming strains F12 and A4, and genome sequencing revealed that only a single gene carried nonsense mutations in both of the strains. In line with a role in gametocyte differentiation, the expression of this gene was predictive for the number of gametocytes that were produced in the two 3D7 clones, so this factor was named PfAP2-G [73,101]. In parallel AP2-G was identified in *P. berghei* non-gametocyte producing cells [72]. Since then, AP2-G has been studied extensively and it has been shown to be a key regulator of gametocytogenesis, meaning that its activation leads to the activation of the differentiation program, which is in line with up to 70% gametocyte conversion rates upon conditional AP2-G overexpression in *P. berghei* [102]. AP2-G contains only one AP2 domain and it is expressed early in sexually committed cells and in stage I gametocytes [33,50,72,73,103]. However, in committed parasites, expression occurs after some other gametocyte specific genes, like GEXP5 [50,104], indicating that AP2-G is not the first factor deciding sexual fate. Nevertheless, it was recently demonstrated that, if expression occurs early enough (before 12 h post invasion in *P. berghei*, or before 20 h post invasion in *P. falciparum*), initiation of the differentiation process is possible within the same cell cycle [102,105]. In non-committed cells, the AP2-G locus is epigenetically silenced but may be stochastically activated [106]. The epigenetic silencing is mediated by PfHda2 (Histone deacetylase 2) and by chromatin binding of the Heterochromatin Protein 1 (HP1), which is removed through a mechanism involving the protein GDV1 (gametocyte development 1) upon commitment [99,107,108,109]. This was also supported by a three-dimensional chromatin confirmation capture study (Hi-C), where the AP2-G locus was found to be located within a cluster of repressed *var* genes in asexual stages, from where it left upon commitment [110]. After de-repression, AP2-G can recognise the (Gx)GTACNC motif in the upstream region of its target genes [34,72,103]. This motif is also eight-fold present in the upstream region (2.1 kb to 3.6 kb) of AP2-G itself, leading to a positive feedback loop that enhances the expression of AP2-G and then triggers the subsequent transcriptional changes [72,73,103,111]. Josling et al., 2019, recently elucidated the target genes of AP2-G by ChIP-seq. As expected, it was found to bind to the promoter regions of many early gametocyte genes. However, surprisingly, it also bound to the promoter regions of invasion genes, especially in committed schizont stage parasites. The binding motif of AP2-G is similar to the GTGCAC motif that was recognised by AP2-I and the direct interaction of AP2-G with AP2-I seems to enhance its function. Among the shared targets of AP2-G and AP2-I, there were also other ApiAP2 TFs, including PF3D7_1239300, SIP2, and PF3D7_1107800. Despite the binding of several invasion related promoters, MSRP1 was the only invasion gene that was specifically upregulated by AP2-G, which may potentially be a reason for reticulocytes being a preferred invasion target for committed merozoites [103]. Interestingly, cooperative interaction between different ApiAP2 TFs was also shown in *T. gondii* between TgAP2XI-5 and TgAP2X-5, which will be discussed later [112].

This type of regulation of the commitment to transmission seems to be conserved in *Plasmodium*, but also in their close relatives, for example, the bovine apicomplexan parasite *Theileria annulata*. In *T. annulata*, it was shown that the orthologue of AP2-G, TaAP2.g (TA13515), binds the same motif GxCTACxC, which is enriched in the upstream regions of genes that were expressed during differentiation of the sexual transmission stages [113]. In addition, the orthologue of the uncharacterised *Plasmodium* ApiAP2 TF PF3D7_0802100, TaAP2.me1 (TA11145), was found to bind to the (A)CACAC(A) motif (the same motif as its orthologue and also the uncharacterised *Plasmodium* ApiAP2 TFs PF3D7_0420300 and PF3D7_1305200), and it probably plays a role in the commitment in *T. annulata*, as it is upregulated during differentiation in comparison to parasites that are incapable of differentiating [113].

In addition to AP2-G, another ApiAP2 TF, AP2-G2, with functions during gametocytogenesis, has been identified in *P. berghei* (PBANKA_1034300). As its name suggests, this factor is believed to function downstream of AP2-G, as knockout nearly completely diminishes gametocyte production (>95%), but, in contrast to AP2-G knockout parasites, these mutants are unable to outgrow their parents [65,72]. Thus, *P. berghei* AP2-G2 knockout parasites still commit to sexual development and their sex, but they mostly fail to mature, and the few parasites that morphologically differentiate into gametocytes cannot exflagellate and cannot be transmitted to mosquitoes [72,76]. In ChIP-Seq analyses, it was shown that AP2-G2 binds to the GTTG(T/C) motif (and the reverse complementary form) of about 1500 predicted target genes, including genes from the asexual, mosquito, and liver stages [76]. Among these genes, the interaction of AP2-G2 with the upstream regions of MSP1 and MSP7 was confirmed by EMSA (electrophoretic mobility shift assay) [76]. Upon knockout of AP2-G2, its target genes were found to be upregulated, suggesting a role for AP2-G2 in gene repression rather than gene activation [76]. Consistent with this proposed function as repressor, the knock out of the *P. berghei* orthologue resulted not only in a block in gametocyte maturation, but also in the derepression of sexual stage genes in schizont stage parasites [65].

Recently, AP2-G3 (PY17X_1417400) has been identified in a CRISPR/Cas9 mediated knock out study in *P. yoelii* as a third gametocyte related ApiAP2 factor [66]. The *P. falciparum* orthologue (PF3D7_1317200) was also implicated in gametogenesis in a piggyback mutagenesis approach, screening for mutations that disrupted the formation of gametocytes [74]. Although its name suggests a role downstream of AP2-G and AP2-G2, it is indeed supposed to act upstream of AP2-G [66]. This is, as, on the one hand, it was shown to be expressed in asexual parasites as well as in gametocytes and it is localised in both the nucleus and in the cytoplasm, so it was proposed to act as a sensor of environmental signals and pose the switch triggering AP2-G regulation [66]. On the other hand, its knockout leads to a loss of gametocyte formation and a decrease in AP2-G expression [66]. Accordingly, a possible hypothesis could be that AP2-G3 senses environmental signals, like homocysteine, LysoPC, and others, and then activates AP2-G, which in turn initiates the differentiation process by activating gametocyte specific genes, while AP2-G2 acts as a repressor for asexual gene expression during the differentiation process. Of note, the *P. vivax* orthologue of AP2-G3, PVX_122680, was implicated in altered gene regulation in developing drug resistance [114,115].

Furthermore, there are several other ApiAP2 TFs that seem to be implicated in gametocytogenesis in *P. falciparum*. Directly after initial AP2-G expression, PF3D7_1222400 is upregulated, and during peak AP2-G expression, the PF3D7_1139300 is also highly expressed [111]. This is in line with the findings of Chaubey et al., who studied the transcriptomic response to ER stress induced by DTT that triggers gametocytogenesis. In this analysis, a cascade of upregulated ApiAP2 TFs was found, with expression of the unknown ApiAP2 PF3D7_1342900 preceding expression of ApiAP2 PF3D7_1139300 and 8 other genes, which was then followed by the upregulation of 23 other genes, including AP2-L (PF3D7_0730300) and ApiAP2 PF3D7_0420300, whereof the latter poses a positive feedback loop for all three other ApiAP2 factors [116]. However, in this cascade of regulated genes, neither AP2-G nor AP2-G2 or AP2-G3 was identified—nevertheless, they reported that the formation of gametocytes was enhanced. This hints to an involvement of metabolic cues for the upregulation of other ApiAP2 factors that again regulate one of the interaction partners of the ApiAP2 genes that leads to an enhancement of AP2-G transcription.

Female gametocytes, as well as the oocysts and sporozoites, were shown to store transcripts that are needed for the later stages [117,118]. This translational repression is mediated, among others, by Puf2 (pumilio family), DOZI (DDX-6 class DEAD box RNA helicase), and CITH (Sm-like factor homolog of CAR-I and Trailer Hitch) [119]. In *P. berghei*, the repressed mRNAs were found to carry a 47bp long, U-rich sequence in the 3′ or 5′ UTR of the mRNA, which is absent in *P. falciparum* [119]. Some ApiAP2 TFs were shown to be already transcribed in gametocytes, but their knockout affects later stages [65,66]. Among those are PF3D7_1305200, PF3D7_1107800, SIP2, AP2-SP2 (PF3D7_0404100), and AP2-O [118]. Of those, AP2-O was downregulated in DOZI KO and CITH KO in *P. berghei* [117].

### 5.3. Gametogenesis and Sporogony

Once the gametocytes are taken up by a mosquito, they differentiate into micro- and macrogametes, which fuse to form the zygote. The zygote then develops into the motile ookinete that crosses the midgut wall and settles on the other side, where finally sporogony is initiated inside the oocyst. These steps are again associated with changes in gene expression, which also involve ApiAP2 TFs [49,120]. The first ookinete specific ApiAP2 that was characterised in *P. berghei* was termed AP2-O (PBANKA_0905900). This factor was essential for the normal morphology of the ookinete and the formation of oocysts [35,66,71]. Interestingly, knockout also attenuated asexual growth, indicating that AP2-O may also play a role during the IDC [65]. AP2-O is transcribed in the female gametocytes and subsequently stored in a complex with the RNA helicase DOZI (development of zygote inhibited) [71,117]. Around 8 h after the fertilisation of the macrogamete, the translation of AP2-O is initiated [71]. AP2-O then activates its target genes, which carry the TAGCTA motif or its reverse complement in their promoter, although similar sequences can also be bound [71]. Genome-wide ChIP analysis revealed that AP2-O targets about 500 genes, which belong to the GO (gene ontology) term categories of pellicle formation and midgut infection, as well as genes that are involved in oocyst development [35].

The knockdown of four other ApiAP2 TFs also had effects from the ookinete stage onwards in *P. berghei* or *P. yoelii*: AP2-O2 (PBANKA_1231600), AP2-O3 (PBANKA_1015500), AP2-O4 (PBANKA_1363700), and AP2_O5 (PY17X_1317000) [65,66]. Of those, ApiAP2-O2 was found to be expressed throughout the asexual cycle and sexual differentiation until the oocyst stage in *P. yoelii* [66]. Knockout of ApiAP2-O2 led to misshaped ookinetes in *P. berghei* but normal ookinetes in *P. yoelii*, respectively; however, both parasite knockout lines were unable to differentiate further [65,66]. In contrast to that, AP2-O3 is not found in asexual stages or ookinetes, but was sex-specifically expressed in female gametocytes as well as in zygotes and oocysts in *P. falciparum* and rodent malaria parasites [66,110]. Its knockout completely abrogates normal ookinetes and further development [65,66,110]. Furthermore, upon AP2-O3 disruption, the genes known to be translationally repressed in female gametocytes were already deregulated in the gametocyte stage [65]. This suggested a role of AP2-O3 in the regulation of genes that are needed for the release of transcriptionally repressed genes [66]. Similar to AP2-O3, AP2-O4 is only expressed in the sexual and mosquito stages; however, its disruption does not affect ookinete development or motility but blocks oocyst and further differentiation [65,66]. Finally, PyAP2-O5 (PY17X_1317000) is expressed in schizonts, gametocytes, and zygotes, but not in ookinetes in *P. yoelii*. The knockout of PyAP2-O5 indicates that it plays a role in ookinete motility and is therefore essential for oocyst development [66].

Inside the oocyst, *Plasmodium* parasites propagate by undergoing sporogony. Sporozoites eventually egress and migrate to the salivary glands. In this stage, specific gene regulation is achieved via ApiAP2 TFs designated AP2-Sp. The first AP2-Sp (PBANKA_1329800) was identified in *P. berghei*. It was shown to be expressed in the late oocyst to sporozoite stage and to recognise the TGCATG(CA) motif with its AP2 domain and adjacent AT-hook [78]. The motif is present in all known sporozoite genes and, in line with that, AP2-Sp knock out leads to a loss of sporogony in the oocyst and the upregulation of gametocyte-specific genes [65,78]. Interestingly, the AP2-Sp motif is also present in the upstream region of PF3D7_0420300, an uncharacterised ApiAP2 factor, suggesting a possible regulatory interaction [78]. Intriguingly, the PbAP2-Sp KO parasites also showed attenuated growth in asexual parasites [65]. As is consistent with a function in asexual stages, PbAP2-Sp is the orthologue of the *P. falciparum* TF that was designated PfAP2-exp, which has a role in the regulation of subtelomeric gene families (see section on asexual stages) [79]. In line with this function it could be shown that in sporozoites generated from clinical isolates from Burkina Faso a single *var* gene, PF3D7_1255200, which carries the DNA motif recognised by PfAP2-EXP, is transcribed [120].

Recently, AP2-Sp2 and AP2-Sp3 were also identified in *P. berghei* and *P. yoelii* [65,66]. AP2-Sp2 was shown to be involved in sporozoite formation in the oocyst, as knockout led to a block of development in the sporoblast stage [65,66]. For the knockout of AP2-Sp3, sporogony was not affected; however, the sporozoites were not motile and it could not exit the oocyst, so they failed to reach the salivary glands and to mature completely [64,65,66]. Interestingly, the orthologue of PbAP2-Sp3 was recently described as PfAP2-Tel in *P. falciparum* (as discussed in Section 5.1). In the future, it will be interesting to investigate whether PbAP2-Sp3 also associates with telomeres in rodent malaria parasites, and whether this may be linked to the observed motility phenotype.

Additionally, further ApiAP2 TFs may be implicated in sporozoite development. The two ApiAP2 TFs PBANKA_0521700/PY17X_0523100 and PBANKA_1319700/PY17X_1323500 are expressed in sporozoites, but the knockout showed no distinct phenotype [66]. However, the *P. falciparum* orthologues both bind the CACACA motif, which possibly indicates the redundancy of these factors [33]. Furthermore, remodelling of the chromatin structure in sporozoites was marked by long-range and interchromosomal contacts of several genes, including the AP2 TF PF3D7_1107800 as well as genes implicated in the liver stage [110].

In *C. parvum*, the ApiAP2 TF cgd4_1110, which is the orthologue of some of the sexual stage-specific factors in *P. falciparum*—like ApiAP2-O2, ApiAP2-O5, ApiAP2-Sp, and others—is not expressed during the asexual part of the life cycle. However, about 48 h to 72 h post infection, when sexual parasites have developed, cgd4_1110 is transcribed, which indicates that it is specifically expressed in the sexual stages [63]. In sporozoites of *Cryptosporidium*, the TFs cgd6_2600 and cgd8_3230, which is orthologous to several sporozoite and oocyst specific *P. falciparum* ApiAP2 TFs, were found to be upregulated [40]. This shows that there seems to be a similar cascade of transcriptional regulation in *Cryptosporidium* sexual stages as in *Plasmodium*, however the complexity is reduced due to the redundancy of the factors.

### 5.4. Liver Stages

Once the sporozoites have reached the liver with the blood circulation, they invade hepatocytes via Kupffer cells, where they start to propagate into merozoites (reviewed in [121]). AP2-L (ortholog of PF3D7_0730300) is the only stage-specific TF that has been so far implicated in this process. AP2-L is expressed in the trophozoites and sporozoites of *P. falciparum* and *P. berghei* [49,70], and the transcript levels are also high in *P. vivax* sporozoites [81]. After liver infection, the PbAP2-L levels increased until 36 h after infection and subsequently decreased again until 48 h after infection [70]. Knockout leads to normal invasion of the sporozoites into the hepatocytes, however their development is disturbed. 24 h after infection, the cells differ in size, and from 36 h after infection onwards, the nuclear division is arrested [65,70]. AP2-L was predicted to recognise the motif AATTTCC and 11 putative target genes, which are mostly implicated in host cell remodelling, could be identified [33,70]. In liver stage parasites, ApiAP2-L was down-regulated by the KO of the sporozoite protein SLARP (Sporozoite and LS asparagine-rich protein) (PF3D7_1147000), which is implicated in liver stage specific transcript storage and stabilisation in the sporozoite, suggesting the translational regulation of AP2-L expression [70,122,123,124]. Another ApiAP2 TF that may possibly be involved in liver stage gene regulation is the uncharacterised PF3D7_1139300, which recognises the TAGAACA motif [125]. This motif was identified in a mutagenesis assays of the promoter sequence of the liver stage specific gene LISP2 (PF3D7_0405300) [125]. The mutation of the motif showed enhanced gene expression, suggesting a potential role of PF3D7_1139300 binding to this motif in gene repression [125].

A few *Plasmodium* species have the capability of forming dormant stages in the liver, which can be reactivated after up to several years of quiescence [126]. In these species, including *P. vivax* or the simian parasite *P. cynomolgi*, another ApiAP2 TF was identified that had no orthologue in *P. falciparum* and the rodent *Plasmodium* species. Therefore this TF was implicated in the biology of the quiescent hypnozoite stages and was named AP2-Q (PCYB_102390, PVP01_1016100). Indeed, a comparison of the transcriptomic data from *P. cynomolgy* hypnozoites and liver schizonts displayed higher expression of AP2-Q in hypnozoites [80], but in another study there was no evidence for the differential regulation of AP2-Q in *P. cynomolgi* [127], and AP2-Q transcripts were only detected at very low levels in these stages in *P. vivax* [128]. However, AP2-Q was transcribed but not translated in sporozoites, indicating that it might be translationally repressed in sporozoites and only translated upon liver infection [81].

Another factor, encoded by PVP01_0916300 was upregulated in *P. vivax* hypnozoites relative to liver schizonts [128], and this TF was also highly expressed in *P. vivax* sporozoites [81]. In contrast, PvAP2-L showed similar expression levels in the dormant and active liver stage parasites [128].

In *P. cynomolgy* hypnozoites, AP2-O2 and AP2-G2 were also upregulated relative to liver schizonts, however the function of these proteins in this stage is unclear [80].

## 6. ApiAP2 Transcription Factors in *Toxoplasma*

The tachyzoite cycle represents the asexual part of the life cycle of *T. gondii*, which can be divided into the G1 and S/M phase. The switch to bradyzoite development occurs in the S/M phase (reviewed by [129]). 24 different ApiAP2 TFs showed distinct expression patterns throughout tachyzoite development, indicating that they may have a role in the timing of gene expression during cell cycle progression, similar to *Plasmodium* [41].

The mutation of the nuclear factor Cactin arrests the parasites in the G1 phase at 40 °C, and transcriptional analysis identified several ApiAP2s that were specifically upregulated, namely TgAP2X-7, TgAP2VIIa-6, TgAP2XII-4, and TgAP2VIII-4 (Table 2) [130]. These data may indicate that these factors are important for suppressing cell cycle progression to S-phase. Another TF, TgAP2XI-5, is expressed throughout the tachyzoite cell cycle and it was implicated in the regulation of virulence genes (among others) during the S/M phase by binding to the palindromic GCTAGC motif [112,131]. Interestingly, TgAP2XI-5 was found to interact with TgAP2X-5, which is most prevalent during the S/M phase of the cell cycle [112]. The disruption of TgAP2X-5 leads to a dramatic decrease in virulence, accompanied by the deregulation of several virulence genes and reduced invasion but normal growth. However, chromatin immunoprecipitation failed to show the direct binding of TgAP2X-5 to the deregulated genes, although TgAP2XI-5 binding to several of the deregulated genes was diminished in the TgAP2X-5 deleted line. This suggests that the cooperation of both factors is needed for efficient regulation—possibly through the formation of heterodimers for motif recognition [112].

Similar to *P. falciparum*, the ApiAP2 TFs of *T. gondii* also interact with chromatin associated factors. Four ApiAP2 TFs (TgAP2IX-7, TgAP2X-8, TgAP2XI-2, and TgAP2XII-4) were co-precipitated with the histone acetyltransferase GCN5b, which is essential for normal parasite growth [132].

In *T. gondii*, the switch from the fast-replicating asexual tachyzoite to the nearly dormant cyst forming bradyzoite, which is often induced by alkaline stress, is essential for transmission to the feline host, where sexual replication takes place. Several ApiAP2 with activating and suppressing functions for this process have been identified. Upon alkaline stress, TgAP2IX-9 is induced [133], which binds the CAGTGT motif [134]. The knockout of TgAP2IX-9 enhanced tissue cyst formation, whereas the overexpression disrupted tissue cyst formation, suggesting a role for TgAP2IX-9 in bradyzoite gene repression [133,134]. In line with this, TgAP2IX-9 was upregulated in a parasite strain that showed higher alkaline stress resistance in comparison to more sensitive isolates [135]. TgAP2IV-4, which is expressed in the late tachyzoite phases, was also associated with a repressive function. While knockout did not affect asexual growth, it lead to the expression of bradyzoite specific genes in tachyzoites [136]. Another ApiAP2 factor, TgAP2IX-4 is dynamically expressed in tachyzoites and early bradyzoites [137]. The knockout of TgAP2IX-4 did not affect tachyzoite proliferation, but lead to the reduced formation of tissue cysts. Transcriptional profiling indicated that TgAP2IX-4 might act via the repression of bradyzoite specifc genes during the early stages of bradyzoites formation, which is possibly restricted to a population of replicating cells within the tissue cyst [137].

After the peak expression of TgAP2IX-9, TgAP2IV-3 is upregulated, which possibly competes for the control of bradyzoite gene expression [133,134]. Later in bradyzoite development, TgAP2XI-4, which induces bradyzoite formation, could replace TgAP2IX-9 [134]. TgAP2XI-4 has been implicated in bradyzoite development and cyst formation upon alkaline stress, as the knockout did not affect tachyzoite growth but inhibited cyst formation [131]. The CACACAC motif is a putative binding site of TgAP2XI-4, and two zinc finger proteins were identified as the possible downstream targets [131].

## 7. Conclusions

The recent publication of numerous studies addressing ApiAP2 function represents a major advance in our understanding of their diverse roles in apicomplexan biology. It is now very well established that ApiAP2s indeed represent the regulatory factors that direct the transcriptional switches that underlie the multiple transitions into morphologically distinct differentiation stages during the life cycles of apicomplexan parasites. ApiAP2 factors clearly have both activating and repressive roles in gene expression, and their interaction with chromatin binding proteins indicates that one of the mechanisms through which they exert regulatory functions is by recruiting the chromatin modifying complexes to target gene promoters [37,86,132]. Highly specific gene regulation, despite some redundancy in binding motifs of ApiAP2 TFs in *Plasmodium* and *Cryptosporidium*, may be achieved by the documented combinatorial action of several ApiAP2 TFs with each other [38,103,112]. The discovery of auto-regulatory loops [73] and the identification of modulatory acetylations in ApiAP2 domains [82] provide mechanisms that contribute to the regulation of ApiAP2 function. In addition to the well documented role of ApiAP2s in gene regulation, some ApiAP2 TFs, namely SIP2 and PfAP2-Tel, have been implicated in chromosome end biology [36,68]. Future investigations are expected to shed light on the molecular mechanisms that underlie the regulatory function of ApiAP2 TFs, for example, how they direct the transcriptional machinery, how they contribute to chromatin remodelling, how they interact with one another to achieve transcriptional control, or how post-translational modifications can modulate their function.

Recently, researchers have begun to exploit the regulatory function of ApiAP2 TFs as a tool for manipulating gene expression in apicomplexan parasites. In one study, engineered promoters carrying ApiAP2 recognition sites (termed “Spooki”) were used to redirect the timing of gene expression in *Plasmodium* ookinetes and sporozoites [138,139]. In another study, the transactivation domains of several AP2 TFs were employed in a tetracycline-dependent system to direct artificial gene regulation in *T. gondii* and *P. berghei* [138,139].

The lack of human orthologues and the essentiality for asexual and sexual differentiation makes ApiAP2 TFs very attractive drug targets. In *T. gondii*, the targeting of AP2XI-3 with phosphorodiamidate morpholine oligomers coupled to transductive peptides that carry small molecules over various membranes inhibited gene expression in a sequence specific manner, resulting in diminished parasite replication in vitro and in vivo in a mouse infection model [140]. Thus, the exploitation of the essential function of ApiAP2 TFs appears as a promising strategy for novel therapeutic approaches.

## Figures and Tables

**Table 1:** ApiAP2 TFs in *Plasmodium* spp.

	*P. falciparum* ApiAP2 TF	Name	Ortholog in *P. vivax*	Ortholog in *P. berghei*	Ortholog in *P. yoelii*	Mutated in *P. falciparum* [67]	Disrupted in *P. berghei*	Disrupted in *P. yoelii* [66]	Phenotype (-| Block)	Motif Recognized [33,34]	Further Ref
1	PF3D7_0404100	AP2-SP2	PVX_001040	PBANKA_1001800	PY17X_1003200	yes	yes [65]	yes	oocyst -| sporoblast		
2	PF3D7_0420300		PVX_090110	PBANKA_0521700	PY17X_0523100	refractory	refractory [64,65]	yes	n.a.	CACACAC, GTGTTACAC	
3	PF3D7_0516800	AP2-O2	PVX_080410	PBANKA_1231600	PY17X_1235000	yes	yes [64,65]	yes	zygote -| ookinete	TGACATCA	
4	PF3D7_0604100	SIP2	PVX_113370	PBANKA_0102900	PY17X_0104500	yes	refractory [64,65]	refractory	n.a.	GGTGCAC	[36]
5	PF3D7_0611200		PVX_113695	PBANKA_0109500	PY17X_0111100	yes	yes [64]	refractory	slow growth		
6	PF3D7_0613800		PVX_113825	PBANKA_0112100	PY17X_0113700	refractory	refractory [64,65]	refractory	n.a.	ATAAGCCCA, CTCTAGAG	
7	PF3D7_0622900	AP2-Tel/SP3	PVX_114260	PBANKA_1121800	PY17X_1123200	yes	yes [65]	yes	sporozoites -| liver stage		[68,69]
8	PF3D7_0730300	AP2-L	PVX_081810	PBANKA_0214400	PY17X_0215800	yes	yes [64,65,70]	refractory	sporozoites -| liver stage	AATTTCC	[70]
9	PF3D7_0802100		n.a.	PBANKA_1228100	PY17X_1231600	refractory	refractory [64,65]	refractory	n.a.	CACACACA	
10	PF3D7_0934400		PVX_086995	PBANKA_0835200	PY17X_0838600	refractory	refractory [64,65]	refractory	n.a.		
11	PF3D7_1007700	AP2-I	PVX_094580	PBANKA_1205900	PY17X_1209100	refractory	refractory [65]	refractory	n.a.	GGGTCGACCC TCTTGCC GTGCACTA	[37]
12	PF3D7_1107800		PVX_091065	PBANKA_0939100	PY17X_0941600	refractory	refractory [64,65]	refractory	n.a.	AGCATAC	
13	PF3D7_1115500		PVX_091420	PBANKA_0932300	PY17X_0934300	refractory	yes [64,65]	refractory	n.a.		
14	PF3D7_1139300		PVX_092570	PBANKA_0909600	PY17X_0911000	yes	refractory [64,65]	refractory	n.a.	TAGAACAA	
15	PF3D7_1143100	AP2-O	PVX_092760	PBANKA_0905900	PY17X_0907300	refractory	yes [64,65,71]	yes	ookinete -| oocyst	TAGCTA [71]	[71]
16	PF3D7_1222400		PVX_123750	n.a.	n.a.	yes	n.a.	n.a.	n.a.	TATATATA	
17	PF3D7_1222600	AP2-G	PVX_123760	PBANKA_1437500	PY17X_1440000	yes	yes [64,65,72]	yes	asexual -| gametocyte	GxGTACxC [72]	[72,73]
18	PF3D7_1239200		PVX_100910	PBANKA_1453700	PY17X_1456200	refractory	refractory [65]	refractory	n.a.	TCTA(C/T)AA	
19	PF3D7_1305200		PVX_122095	PBANKA_1403700	PY17X_1405400	yes	refractory [64,65]	refractory	n.a.	TGCACACAC	
20	PF3D7_1317200	AP2-G3	PVX_122680	PBANKA_1415700	PY17X_1417400	yes	refractory [65]	yes	asexual -| gametocyte	TAGCTCA(G/A)A	[74]
21	PF3D7_1342900		PVX_083040	PBANKA_1356000	PY17X_1361700	yes	refractory [65]	refractory	n.a.	GCGGGGC	[69]
22	PF3D7_1350900	AP2-O4	PVX_083440	PBANKA_1363700	PY17X_1369400	refractory	yes [64,65]	yes	ookinete -| oocyst	ATTCTAGAA	[75]
23	PF3D7_1408200	AP2-G2	PVX_086035	PBANKA_1034300	PY17X_1036700	yes	yes [64,65,72,76]	yes	asexual -| gametocyte	GTTG(T/C) [76]	[72,76]
24	PF3D7_1429200	AP2-O3	PVX_085085	PBANKA_1015500	PY17X_1017000	yes	yes [65]	yes	zygote -| ookinete		[77]
25	PF3D7_1449500	AP2-O5	PVX_118015	PBANKA_1313200	PY17X_1317000	refractory	refractory [64]	yes	zygote -| ookinete		
26	PF3D7_1456000		PVX_117665	PBANKA_1319700	PY17X_1323500	yes	yes [64,65]	yes	dispensable	CACACACAC	
27	PF3D7_1466400	AP2-SP/EXP	PVX_117145	PBANKA_1329800	PY17X_1334500	yes	yes [64,65,78]	refractory	oocyst -| sporoblast	TGCATGCA	[31,79]
28	n.a.	AP2-Q	PVX_080355	n.a.	n.a.	n.a.	n.a.	n.a.	n.a.		[80,81]

**Table 2:** ApiAP2 TFs in *T. gondii*.

	AP2	Name	Function/Phenotype	Interactions
1	TGME49_208020	AP2Ib-1		
2	TGME49_252370	AP2III-1		
3	TGME49_253380	AP2III-2		
4	TGME49_299150	AP2III-3		
5	TGME49_299020	AP2III-4		
6	TGME49_320700	AP2IV-1		
7	TGME49_320680	AP2IV-2		
8	TGME49_318610	AP2IV-3	Control of bradyzoite genes [133,134]	
9	TGME49_318470	AP2IV-4	KO leads to expression of bradyzoite specific genes in tachyzoites [136]	
10	TGME49_211720	AP2IV-5		
11	TGME49_267460	AP2IX-1		
12	TGME49_264485	AP2IX-3		
13	TGME49_288950	AP2IX-4	KO leads to reduces tissue cyst formation [137]	
14	TGME49_289710	AP2IX-5		
15	TGME49_290180	AP2IX-6		
16	TGME49_290630	AP2IX-7	Implicated in cell cycle progression to S-phase [130]	GCN5b [132]
17	TGME49_306000	AP2IX-8		
18	TGME49_306620	AP2IX-9	KO enhances cyst formation, alkaline stress response [133,134]	
19	TGME49_220530	AP2V-1		
20	TGME49_285895	AP2V-2		
21	TGME49_240460	AP2VI-1		
22	TGME49_240900	AP2VI-2		
23	TGME49_244510	AP2VI-3		
24	TGME49_280470	AP2VIIa-1		
25	TGME49_280460	AP2VIIa-2		
26	TGME49_205650	AP2VIIa-3		
27	TGME49_203710	AP2VIIa-4		
28	TGME49_203690	AP2VIIa-5		
29	TGME49_203050	AP2VIIa-6	Implicated in cell cycle progression to S-phase [130]	
30	TGME49_202490	AP2VIIa-7		
31	TGME49_282210	AP2VIIa-8		
32	TGME49_282220	AP2VIIa-9		
33	TGME49_262420	APVIIb-1/ADA2-B		
34	TGME49_262000	AP2VIIb-2		
35	TGME49_255220	AP2VIIb-3		
36	TGME49_229370	AP2VIII-1		
37	TGME49_233120	AP2VIII-2		
38	TGME49_273660	AP2VIII-3		
39	TGME49_272710	AP2VIII-4	Implicated in cell cycle progression to S-phase [130]	
40	TGME49_271200	AP2VIII-5		
41	TGME49_271030	AP2VIII-6		
42	TGME49_269010	AP2VIII-7		
43	TGME49_227900	AP2X-1		
44	TGME49_225110	AP2X-2		
45	TGME49_224230	AP2X-3		
46	TGME49_224050	AP2X-4		
47	TGME49_237090	AP2X-5	Implicated in virulence gene regulation [112]	AP2XI-5 [112]
48	TGME49_237425	AP2X-6		
49	TGME49_214840	AP2X-7	Implicated in cell cycle progression to S-phase [130]	
50	TGME49_214960	AP2X-8		GCN5b [132]
51	TGME49_215150	AP2X-9		
52	TGME49_215340	AP2X-10		
53	TGME49_215570	AP2X-11		
54	TGME49_309410	AP2XI-1		
55	TGME49_310900	AP2XI-2		GCN5b [132]
56	TGME49_310950	AP2XI-3		
57	TGME49_315760	AP2XI-4	KO inhibits cyst formation [131,134]	
58	TGME49_216220	AP2XI-5	Implicated in virulence gene regulation [112]	AP2X-5 [112]
59	TGME49_215895	AP2 domain-containing protein		
60	TGME49_218960	AP2XII-1		
61	TGME49_217700	AP2XII-2		
62	TGME49_246660	AP2XII-3		
63	TGME49_247700	AP2XII-4	Implicated in cell cycle progression to S-phase [130]	GCN5b [132]
64	TGME49_247730	AP2XII-5		
65	TGME49_249190	AP2XII-6		
66	TGME49_250800	AP2XII-8		
67	TGME49_251740	AP2XII-9		

## References

[1] WHO (2017). WHO Malaria Report 2017.

[2] Montoya J.G., Liesenfeld O. (2004). Toxoplasmosis. Lancet.

[3] Khalil I.A., Troeger C., Rao P.C., Blacker B.F., Brown A., Brewer T.G., Colombara D.V., De Hostos E.L., Engmann C., Guerrant R.L. (2018). Morbidity, mortality, and long-term consequences associated with diarrhoea from *Cryptosporidium* infection in children younger than 5 years: A meta-analyses study. Lancet Glob. Health.

[4] Menard D., Dondorp A. (2017). Antimalarial Drug Resistance: A Threat to Malaria Elimination. Cold Spring Harb. Perspect. Med..

[5] Montazeri M., Mehrzadi S., Sharif M., Sarvi S., Tanzifi A., Aghayan S.A., Daryani A. (2018). Drug Resistance in Toxoplasma gondii. Front. Microbiol..

[6] Bozdech Z., Llinás M., Pulliam B.L., Wong E.D., Zhu J., DeRisi J.L. (2003). The Transcriptome of the Intraerythrocytic Developmental Cycle of Plasmodium falciparum. PLoS Biol..

[7] Le Roch K.G., Zhou Y., Blair P.L., Grainger M., Moch J.K., Haynes J.D., De La Vega P., Holder A.A., Batalov S., Carucci D.J. (2003). Discovery of gene function by expression profiling of the malaria parasite life cycle. Science.

[8] Radke J.R., Behnke M.S., Mackey A.J., Radke J.B., Roos D.S., White M.W. (2005). The transcriptome of Toxoplasma gondii. BMC Biol..

[9] Behnke M.S., Radke J.B., Smith A.T., Sullivan W.J., White M.W. (2008). The transcription of bradyzoite genes in Toxoplasma gondii is controlled by autonomous promoter elements. Mol. Microbiol..

[10] Lopez-Barragan M.J., Lemieux J., Quinones M., Williamson K.C., Molina-Cruz A., Cui K., Barillas-Mury C., Zhao K., Su X.Z. (2011). Directional gene expression and antisense transcripts in sexual and asexual stages of Plasmodium falciparum. BMC Genom..

[11] Duffy M.F., Selvarajah S.A., Josling G.A., Petter M. (2014). Epigenetic regulation of the Plasmodium falciparum genome. Brief. Funct. Genom..

[12] Latchman D.S. (1997). Transcription factors: An overview. Int. J. Biochem. Cell Biol..

[13] Aravind L., Iyer L.M., Wellems T.E., Miller L.H. (2003). Plasmodium Biology: Genomic Gleanings. Cell.

[14] Templeton T.J., Iyer L.M., Anantharaman V., Enomoto S., Abrahante J.E., Subramanian G.M., Hoffman S.L., Abrahamsen M.S., Aravind L. (2004). Comparative analysis of apicomplexa and genomic diversity in eukaryotes. Genome Res..

[15] Coulson R.M.R., Hall N., Ouzounis C.A. (2004). Comparative genomics of transcriptional control in the human malaria parasite Plasmodium falciparum. Genome Res..

[16] Boschet C., Gissot M., Briquet S., Hamid Z., Claudel-Renard C., Vaquero C. (2004). Characterization of PfMyb1 transcription factor during erythrocytic development of 3D7 and F12 Plasmodium falciparum clones. Mol. Biochem. Parasitol..

[17] Gissot M., Ting L.M., Daly T.M., Bergman L.W., Sinnis P., Kim K. (2008). High mobility group protein HMGB2 is a critical regulator of plasmodium oocyst development. J. Biol. Chem..

[18] Briquet S., Boschet C., Gissot M., Tissandie E., Sevilla E., Franetich J.F., Thiery I., Hamid Z., Bourgouin C., Vaquero C. (2006). High-mobility-group box nuclear factors of Plasmodium falciparum. Eukaryot. Cell.

[19] Iyer L.M., Anantharaman V., Wolf M.Y., Aravind L. (2008). Comparative genomics of transcription factors and chromatin proteins in parasitic protists and other eukaryotes. Int. J. Parasitol..

[20] Komaki-Yasuda K., Okuwaki M., Nagata K., Kawazu S., Kano S. (2013). Identification of a novel and unique transcription factor in the intraerythrocytic stage of Plasmodium falciparum. PLoS ONE.

[21] Balaji S., Babu M.M., Iyer L.M., Aravind L. (2005). Discovery of the principal specific transcription factors of Apicomplexa and their implication for the evolution of the AP2-integrase DNA binding domains. Nucleic Acids Res..

[22] Yamasaki K., Kigawa T., Seki M., Shinozaki K., Yokoyama S. (2013). DNA-binding domains of plant-specific transcription factors: Structure, function, and evolution. Trends Plant Sci..

[23] McFadden G.I., Reith M.E., Munholland J., Lang-Unnasch N. (1996). Plastid in human parasites. Nature.

[24] Waller R.F., McFadden G.I. (2005). The apicoplast: A review of the derived plastid of apicomplexan parasites. Curr. Issues Mol. Biol..

[25] Janouskovec J., Horak A., Obornik M., Lukes J., Keeling P.J. (2010). A common red algal origin of the apicomplexan, dinoflagellate, and heterokont plastids. Proc. Natl. Acad. Sci. USA.

[26] Foth B.J., McFadden G.I. (2003). The apicoplast: A plastid in Plasmodium falciparum and other Apicomplexan parasites. Int. Rev. Cytol..

[27] Aurrecoechea C., Barreto A., Basenko E.Y., Brestelli J., Brunk B.P., Cade S., Crouch K., Doherty R., Falke D., Fischer S. (2017). EuPathDB: The eukaryotic pathogen genomics database resource. Nucleic Acids Res..

[28] Oberstaller J., Pumpalova Y., Schieler A., Llinas M., Kissinger J.C. (2014). The Cryptosporidium parvum ApiAP2 gene family: Insights into the evolution of apicomplexan AP2 regulatory systems. Nucleic Acids Res..

[29] Oberstaller J., Joseph S.J., Kissinger J.C. (2013). Genome-wide upstream motif analysis of Cryptosporidium parvum genes clustered by expression profile. BMC Genom..

[30] Oehring S.C., Woodcroft B.J., Moes S., Wetzel J., Dietz O., Pulfer A., Dekiwadia C., Maeser P., Flueck C., Witmer K. (2012). Organellar proteomics reveals hundreds of novel nuclear proteins in the malaria parasite Plasmodium falciparum. Genome Biol..

[31] Lindner S.E., De Silva E.K., Keck J.L., Llinás M. (2010). Structural determinants of DNA binding by a P. falciparum ApiAP2 transcriptional regulator. J. Mol. Biol..

[32] Aravind L., Landsman D. (1998). AT-hook motifs identified in a wide variety of DNA-binding proteins. Nucleic Acids Res..

[33] Campbell T.L., De Silva E.K., Olszewski K.L., Elemento O., Llinas M. (2010). Identification and genome-wide prediction of DNA binding specificities for the ApiAP2 family of regulators from the malaria parasite. PLoS Pathog..

[34] De Silva E.K., Gehrke A.R., Olszewski K., Leon I., Chahal J.S., Bulyk M.L., Llinas M. (2008). Specific DNA-binding by apicomplexan AP2 transcription factors. Proc. Natl. Acad. Sci. USA.

[35] Kaneko I., Iwanaga S., Kato T., Kobayashi I., Yuda M. (2015). Genome-Wide Identification of the Target Genes of AP2-O, a Plasmodium AP2-Family Transcription Factor. PLoS Pathog..

[36] Flueck C., Bartfai R., Niederwieser I., Witmer K., Alako B.T., Moes S., Bozdech Z., Jenoe P., Stunnenberg H.G., Voss T.S. (2010). A major role for the Plasmodium falciparum ApiAP2 protein PfSIP2 in chromosome end biology. PLoS Pathog..

[37] Santos J.M., Josling G., Ross P., Joshi P., Orchard L., Campbell T., Schieler A., Cristea I.M., Llinas M. (2017). Red Blood Cell Invasion by the Malaria Parasite Is Coordinated by the PfAP2-I Transcription Factor. Cell Host Microbe.

[38] Toenhake C.G., Fraschka S.A., Vijayabaskar M.S., Westhead D.R., van Heeringen S.J., Bartfai R. (2018). Chromatin Accessibility-Based Characterization of the Gene Regulatory Network Underlying Plasmodium falciparum Blood-Stage Development. Cell Host Microbe.

[39] Ruiz J.L., Tena J.J., Bancells C., Cortes A., Gomez-Skarmeta J.L., Gomez-Diaz E. (2018). Characterization of the accessible genome in the human malaria parasite Plasmodium falciparum. Nucleic Acids Res..

[40] Lippuner C., Ramakrishnan C., Basso W.U., Schmid M.W., Okoniewski M., Smith N.C., Hässig M., Deplazes P., Hehl A.B. (2018). RNA-Seq analysis during the life cycle of Cryptosporidium parvum reveals significant differential gene expression between proliferating stages in the intestine and infectious sporozoites. Int. J. Parasitol..

[41] Behnke M.S., Wootton J.C., Lehmann M.M., Radke J.B., Lucas O., Nawas J., Sibley L.D., White M.W. (2010). Coordinated progression through two subtranscriptomes underlies the tachyzoite cycle of Toxoplasma gondii. PLoS ONE.

[42] Behnke M.S., Zhang T.P., Dubey J.P., Sibley L.D. (2014). Toxoplasma gondii merozoite gene expression analysis with comparison to the life cycle discloses a unique expression state during enteric development. BMC Genom..

[43] Gaji R.Y., Behnke M.S., Lehmann M.M., White M.W., Carruthers V.B. (2011). Cell cycle-dependent, intercellular transmission of Toxoplasma gondii is accompanied by marked changes in parasite gene expression. Mol. Microbiol..

[44] Mirhashemi M.E., Noubary F., Chapman-Bonofiglio S., Tzipori S., Huggins G.S., Widmer G. (2018). Transcriptome analysis of pig intestinal cell monolayers infected with Cryptosporidium parvum asexual stages. Parasites Vectors.

[45] Pelle K.G., Oh K., Buchholz K., Narasimhan V., Joice R., Milner D.A., Brancucci N.M., Ma S., Voss T.S., Ketman K. (2015). Transcriptional profiling defines dynamics of parasite tissue sequestration during malaria infection. Genome Med..

[46] Lasonder E., Rijpma S.R., van Schaijk B.C., Hoeijmakers W.A., Kensche P.R., Gresnigt M.S., Italiaander A., Vos M.W., Woestenenk R., Bousema T. (2016). Integrated transcriptomic and proteomic analyses of P. falciparum gametocytes: Molecular insight into sex-specific processes and translational repression. Nucleic Acids Res..

[47] Otto T.D., Wilinski D., Assefa S., Keane T.M., Sarry L.R., Bohme U., Lemieux J., Barrell B., Pain A., Berriman M. (2010). New insights into the blood-stage transcriptome of Plasmodium falciparum using RNA-Seq. Mol. Microbiol..

[48] Bartfai R., Hoeijmakers W.A., Salcedo-Amaya A.M., Smits A.H., Janssen-Megens E., Kaan A., Treeck M., Gilberger T.W., Francoijs K.J., Stunnenberg H.G. (2010). H2A.Z demarcates intergenic regions of the plasmodium falciparum epigenome that are dynamically marked by H3K9ac and H3K4me3. PLoS Pathog..

[49] Zanghi G., Vembar S.S., Baumgarten S., Ding S., Guizetti J., Bryant J.M., Mattei D., Jensen A.T.R., Renia L., Goh Y.S. (2018). A Specific PfEMP1 Is Expressed in P. falciparum Sporozoites and Plays a Role in Hepatocyte Infection. Cell Rep..

[50] Painter H.J., Carrasquilla M., Llinás M. (2017). Capturing in vivo RNA transcriptional dynamics from the malaria parasite Plasmodium falciparum. Genome Res..

[51] Painter H.J., Chung N.C., Sebastian A., Albert I., Storey J.D., Llinás M. (2018). Genome-wide real-time in vivo transcriptional dynamics during Plasmodium falciparum blood-stage development. Nat. Commun..

[52] Bunnik E.M., Chung D.W., Hamilton M., Ponts N., Saraf A., Prudhomme J., Florens L., Le Roch K.G. (2013). Polysome profiling reveals translational control of gene expression in the human malaria parasite Plasmodium falciparum. Genome Biol..

[53] Buchholz K.R., Fritz H.M., Chen X., Durbin-Johnson B., Rocke D.M., Ferguson D.J., Conrad P.A., Boothroyd J.C. (2011). Identification of tissue cyst wall components by transcriptome analysis of in vivo and in vitro Toxoplasma gondii bradyzoites. Eukaryot. Cell.

[54] Fritz H.M., Buchholz K.R., Chen X., Durbin-Johnson B., Rocke D.M., Conrad P.A., Boothroyd J.C. (2012). Transcriptomic analysis of toxoplasma development reveals many novel functions and structures specific to sporozoites and oocysts. PLoS ONE.

[55] Mota M.M., Rodriguez A. (2002). Invasion of mammalian host cells by Plasmodium sporozoites. Bioessays News Rev. Mol. Cell. Dev. Biol..

[56] Field J.W., Shute P.G. (1956). The Microscopic Diagnosis of Human Malaria II.

[57] Silvestrini F., Alano P., Williams J.L. (2000). Commitment to the production of male and female gametocytes in the human malaria parasite Plasmodium falciparum. Parasitology.

[58] Bennink S., Kiesow M.J., Pradel G. (2016). The development of malaria parasites in the mosquito midgut. Cell. Microbiol..

[59] Matuschewski K. (2006). Getting infectious: Formation and maturation of Plasmodium sporozoites in the Anopheles vector. Cell. Microbiol..

[60] Baton L.A., Ranford-Cartwright L.C. (2005). How do malaria ookinetes cross the mosquito midgut wall?. Trends Parasitol..

[61] Gubbels M.J., White M., Szatanek T. (2008). The cell cycle and Toxoplasma gondii cell division: Tightly knit or loosely stitched?. Int. J. Parasitol..

[62] Bouzid M., Hunter P.R., Chalmers R.M., Tyler K.M. (2013). Cryptosporidium pathogenicity and virulence. Clin. Microbiol. Rev..

[63] Mauzy M.J., Enomoto S., Lancto C.A., Abrahamsen M.S., Rutherford M.S. (2012). The Cryptosporidium parvum transcriptome during in vitro development. PLoS ONE.

[64] Bushell E., Gomes A.R., Sanderson T., Anar B., Girling G., Herd C., Metcalf T., Modrzynska K., Schwach F., Martin R.E. (2017). Functional Profiling of a Plasmodium Genome Reveals an Abundance of Essential Genes. Cell.

[65] Modrzynska K., Pfander C., Chappell L., Yu L., Suarez C., Dundas K., Gomes A.R., Goulding D., Rayner J.C., Choudhary J. (2017). A Knockout Screen of ApiAP2 Genes Reveals Networks of Interacting Transcriptional Regulators Controlling the Plasmodium Life Cycle. Cell Host Microbe.

[66] Zhang C., Li Z., Cui H., Jiang Y., Yang Z., Wang X., Gao H., Liu C., Zhang S., Su X.-Z. (2017). Systematic CRISPR-Cas9-Mediated Modifications of Plasmodium yoelii ApiAP2 Genes Reveal Functional Insights into Parasite Development. mBio.

[67] Zhang M., Wang C., Otto T.D., Oberstaller J., Liao X., Adapa S.R., Udenze K., Bronner I.F., Casandra D., Mayho M. (2018). Uncovering the essential genes of the human malaria parasite Plasmodium falciparum by saturation mutagenesis. Science.

[68] Sierra-Miranda M., Vembar S.-S., Delgadillo D.M., Ávila-López P.A., Herrera-Solorio A.-M., Lozano Amado D., Vargas M., Hernandez-Rivas R. (2017). PfAP2Tel, harbouring a non-canonical DNA-binding AP2 domain, binds to Plasmodium falciparum telomeres. Cell. Microbiol..

[69] Balu B., Singh N., Maher S.P., Adams J.H. (2010). A genetic screen for attenuated growth identifies genes crucial for intraerythrocytic development of Plasmodium falciparum. PLoS ONE.

[70] Iwanaga S., Kaneko I., Kato T., Yuda M. (2012). Identification of an AP2-family protein that is critical for malaria liver stage development. PLoS ONE.

[71] Yuda M., Iwanaga S., Shigenobu S., Mair G.R., Janse C.J., Waters A.P., Kato T., Kaneko I. (2009). Identification of a transcription factor in the mosquito-invasive stage of malaria parasites. Mol. Microbiol..

[72] Sinha A., Hughes K.R., Modrzynska K.K., Otto T.D., Pfander C., Dickens N.J., Religa A.A., Bushell E., Graham A.L., Cameron R. (2014). A cascade of DNA-binding proteins for sexual commitment and development in Plasmodium. Nature.

[73] Kafsack B.F., Rovira-Graells N., Clark T.G., Bancells C., Crowley V.M., Campino S.G., Williams A.E., Drought L.G., Kwiatkowski D.P., Baker D.A. (2014). A transcriptional switch underlies commitment to sexual development in malaria parasites. Nature.

[74] Ikadai H., Shaw Saliba K., Kanzok S.M., McLean K.J., Tanaka T.Q., Cao J., Williamson K.C., Jacobs-Lorena M. (2013). Transposon mutagenesis identifies genes essential for Plasmodium falciparum gametocytogenesis. Proc. Natl. Acad. Sci. USA.

[75] Bischoff E., Vaquero C. (2010). In silico and biological survey of transcription-associated proteins implicated in the transcriptional machinery during the erythrocytic development of Plasmodium falciparum. BMC Genom..

[76] Yuda M., Iwanaga S., Kaneko I., Kato T. (2015). Global transcriptional repression: An initial and essential step for Plasmodium sexual development. PNAS.

[77] Maier A.G., Rug M., O’Neill M.T., Brown M., Chakravorty S., Szestak T., Chesson J., Wu Y., Hughes K., Coppel R.L. (2008). Exported proteins required for virulence and rigidity of Plasmodium falciparum-infected human erythrocytes. Cell.

[78] Yuda M., Iwanaga S., Shigenobu S., Kato T., Kaneko I. (2010). Transcription factor AP2-Sp and its target genes in malarial sporozoites. Mol. Microbiol..

[79] Martins R.M., Macpherson C.R., Claes A., Scheidig-Benatar C., Sakamoto H., Yam X.Y., Preiser P., Goel S., Wahlgren M., Sismeiro O. (2017). An ApiAP2 member regulates expression of clonally variant genes of the human malaria parasite Plasmodium falciparum. Sci. Rep..

[80] Cubi R., Vembar S.S., Biton A., Franetich J.-F., Bordessoulles M., Sossau D., Zanghi G., Bosson-Vanga H., Benard M., Moreno A. (2017). Laser capture microdissection enables transcriptomic analysis of dividing and quiescent liver stages of Plasmodium relapsing species. Cell. Microbiol..

[81] Jex A., Mueller I., Kappe S., Mikolajcjak S., Sattabongkot J., Patrapuvich R., Lindner S., Flannery E., Koepfli C., Ansell B. (2019). Transcriptome and histone epigenome of *Plasmodium vivax* salivary-gland sporozoites point to tight regulatory control and potential mechanisms for liver-stage differentiation. Int. J. Parasitol..

[82] Cobbold S.A., Santos J.M., Ochoa A., Perlman D.H., Llinas M. (2016). Proteome-wide analysis reveals widespread lysine acetylation of major protein complexes in the malaria parasite. Sci. Rep..

[83] Young J.A., Johnson J.R., Benner C., Yan S.F., Chen K., Le Roch K.G., Zhou Y., Winzeler E.A. (2008). In silico discovery of transcription regulatory elements in Plasmodium falciparum. BMC Genom..

[84] Iengar P., Joshi N.V. (2009). Identification of putative regulatory motifs in the upstream regions of co-expressed functional groups of genes in Plasmodium falciparum. BMC Genom..

[85] Essien K., Stoeckert C.J. (2010). Conservation and divergence of known apicomplexan transcriptional regulons. BMC Genom..

[86] Josling G.A., Petter M., Oehring S.C., Gupta A.P., Dietz O., Wilson D.W., Schubert T., Langst G., Gilson P.R., Crabb B.S. (2015). A Plasmodium Falciparum Bromodomain Protein Regulates Invasion Gene Expression. Cell Host Microbe.

[87] Cowell A., Winzeler E. (2018). Exploration of the Plasmodium falciparum Resistome and Druggable Genome Reveals New Mechanisms of Drug Resistance and Antimalarial Targets. Microbiol. Insights.

[88] Wendler J.P., Okombo J., Amato R., Miotto O., Kiara S.M., Mwai L., Pole L., O’Brien J., Manske M., Alcock D. (2014). A genome wide association study of Plasmodium falciparum susceptibility to 22 antimalarial drugs in Kenya. PLoS ONE.

[89] van Biljon R., Niemand J., van Wyk R., Clark K., Verlinden B., Abrie C., von Grüning H., Smidt W., Smit A., Reader J. (2018). Inducing controlled cell cycle arrest and re-entry during asexual proliferation of Plasmodium falciparum malaria parasites. Sci. Rep..

[90] Bankier A.T., Spriggs H.F., Fartmann B., Konfortov B.A., Madera M., Vogel C., Teichmann S.A., Ivens A., Dear P.H. (2003). Integrated mapping, chromosomal sequencing and sequence analysis of Cryptosporidium parvum. Genome Res..

[91] Ling K.-H., Rajandream M.-A., Rivailler P., Ivens A., Yap S.-J., Madeira A.M.B.N., Mungall K., Billington K., Yee W.-Y., Bankier A.T. (2007). Sequencing and analysis of chromosome 1 of Eimeria tenella reveals a unique segmental organization. Genome Res..

[92] Voss T.S., Kaestli M., Vogel D., Bopp S., Beck H.P. (2003). Identification of nuclear proteins that interact differentially with Plasmodium falciparum var gene promoters. Mol. Microbiol..

[93] Rono M.K., Nyonda M.A., Simam J.J., Ngoi J.M., Mok S., Kortok M.M., Abdullah A.S., Elfaki M.M., Waitumbi J.N., El-Hassan I.M. (2018). Adaptation of Plasmodium falciparum to its transmission environment. Nat. Ecol. Evol..

[94] Honma H., Niikura M., Kobayashi F., Horii T., Mita T., Endo H., Hirai M. (2016). Mutation tendency of mutator Plasmodium berghei with proofreading-deficient DNA polymerase δ. Sci. Rep..

[95] Claessens A., Affara M., Assefa S.A., Kwiatkowski D.P., Conway D.J. (2017). Culture adaptation of malaria parasites selects for convergent loss-of-function mutants. Sci. Rep..

[96] Beri D., Balan B., Chaubey S., Subramaniam S., Surendra B., Tatu U. (2017). A disrupted transsulphuration pathway results in accumulation of redox metabolites and induction of gametocytogenesis in malaria. Sci. Rep..

[97] Brancucci N.M.B., Gerdt J.P., Wang C., De Niz M., Philip N., Adapa S.R., Zhang M., Hitz E., Niederwieser I., Boltryk S.D. (2017). Lysophosphatidylcholine Regulates Sexual Stage Differentiation in the Human Malaria Parasite Plasmodium falciparum. Cell.

[98] Rovira-Graells N., Gupta A.P., Planet E., Crowley V.M., Mok S., Ribas de Pouplana L., Preiser P.R., Bozdech Z., Cortés A. (2012). Transcriptional variation in the malaria parasite Plasmodium falciparum. Genome Res..

[99] Eksi S., Morahan B.J., Haile Y., Furuya T., Jiang H., Ali O., Xu H., Kiattibutr K., Suri A., Czesny B. (2012). Plasmodium falciparum gametocyte development 1 (Pfgdv1) and gametocytogenesis early gene identification and commitment to sexual development. PLoS Pathog..

[100] Day K.P., Karamalis F., Thompson J., Barnes D.A., Peterson C., Brown H., Brown G.V., Kemp D.J. (1993). Genes necessary for expression of a virulence determinant and for transmission of Plasmodium falciparum are located on a 0.3-megabase region of chromosome 9. Proc. Natl. Acad. Sci. USA.

[101] Campino S., Benavente E.D., Assefa S., Thompson E., Drought L.G., Taylor C.J., Gorvett Z., Carret C.K., Flueck C., Ivens A.C. (2016). Genomic variation in two gametocyte non-producing Plasmodium falciparum clonal lines. Malar. J..

[102] Kent R.S., Modrzynska K.K., Cameron R., Philip N., Billker O., Waters A.P. (2018). Inducible developmental reprogramming redefines commitment to sexual development in the malaria parasite Plasmodium berghei. Nat. Microbiol..

[103] Josling G.A., Venezia J., Orchard L., Russell T.J., Painter H.J., Llinas M. (2019). Regulation of sexual differentiation is linked to invasion in malaria parasites. bioRxiv.

[104] Tiburcio M., Dixon M.W., Looker O., Younis S.Y., Tilley L., Alano P. (2015). Specific expression and export of the Plasmodium falciparum Gametocyte EXported Protein-5 marks the gametocyte ring stage. Malar J.

[105] Bancells C., Llorà-Batlle O., Poran A., Nötzel C., Rovira-Graells N., Elemento O., Kafsack B.F.C., Cortés A. (2019). Revisiting the initial steps of sexual development in the malaria parasite Plasmodium falciparum. Nat. Microbiol..

[106] Cortés A., Crowley V.M., Vaquero A., Voss T.S. (2012). A View on the Role of Epigenetics in the Biology of Malaria Parasites. PLoS Pathog..

[107] Coleman B.I., Skillman K.M., Jiang R.H.Y., Childs L.M., Altenhofen L.M., Ganter M., Leung Y., Goldowitz I., Kafsack B.F.C., Marti M. (2014). A Plasmodium falciparum histone deacetylase regulates antigenic variation and gametocyte conversion. Cell Host Microbe.

[108] Brancucci N.M.B., Bertschi N.L., Zhu L., Niederwieser I., Chin W.H., Wampfler R., Freymond C., Rottmann M., Felger I., Bozdech Z. (2014). Heterochromatin protein 1 secures survival and transmission of malaria parasites. Cell Host Microbe.

[109] Filarsky M., Fraschka S.A., Niederwieser I., Brancucci N.M.B., Carrington E., Carrió E., Moes S., Jenoe P., Bártfai R., Voss T.S. (2018). GDV1 induces sexual commitment of malaria parasites by antagonizing HP1-dependent gene silencing. Science.

[110] Bunnik E.M., Cook K.B., Varoquaux N., Batugedara G., Prudhomme J., Cort A., Shi L., Andolina C., Ross L.S., Brady D. (2018). Changes in genome organization of parasite-specific gene families during the Plasmodium transmission stages. Nat. Commun..

[111] Poran A., Notzel C., Aly O., Mencia-Trinchant N., Harris C.T., Guzman M.L., Hassane D.C., Elemento O., Kafsack B.F.C. (2017). Single-cell RNA sequencing reveals a signature of sexual commitment in malaria parasites. Nature.

[112] Lesage K.M., Huot L., Mouveaux T., Courjol F., Saliou J.-M., Gissot M. (2018). Cooperative binding of ApiAP2 transcription factors is crucial for the expression of virulence genes in Toxoplasma gondii. Nucleic Acids Res..

[113] Pieszko M., Weir W., Goodhead I., Kinnaird J., Shiels B. (2015). ApiAP2 Factors as Candidate Regulators of Stochastic Commitment to Merozoite Production in Theileria annulata. PLoS Negl. Trop. Dis..

[114] Parobek C.M., Lin J.T., Saunders D.L., Barnett E.J., Lon C., Lanteri C.A., Balasubramanian S., Brazeau N., DeConti D.K., Garba D.L. (2016). Selective sweep suggests transcriptional regulation may underlie Plasmodium vivax resilience to malaria control measures in Cambodia. Proc. Natl. Acad. Sci. USA.

[115] Dharia N.V., Bright A.T., Westenberger S.J., Barnes S.W., Batalov S., Kuhen K., Borboa R., Federe G.C., McClean C.M., Vinetz J.M. (2010). Whole-genome sequencing and microarray analysis of ex vivo Plasmodium vivax reveal selective pressure on putative drug resistance genes. Proc. Natl. Acad. Sci. USA.

[116] Chaubey S., Grover M., Tatu U. (2014). Endoplasmic reticulum stress triggers gametocytogenesis in the malaria parasite. J. Biol. Chem..

[117] Mair G.R., Lasonder E., Garver L.S., Franke-Fayard B.M.D., Carret C.K., Wiegant J.C.A.G., Dirks R.W., Dimopoulos G., Janse C.J., Waters A.P. (2010). Universal features of post-transcriptional gene regulation are critical for Plasmodium zygote development. PLoS Pathog..

[118] Mair G.R., Braks J.A.M., Garver L.S., Dimopoulos G., Hall N., Wiegant J.C.A.G., Dirks R.W., Khan S.M., Janse C.J., Waters A.P. (2006). Translational Repression is essential for Plasmodium sexual development and mediated by a DDX6-type RNA helicase. Science.

[119] Cui L., Lindner S., Miao J. (2015). Translational regulation during stage transitions in malaria parasites. Ann. N. Y. Acad. Sci..

[120] Gomez-Diaz E., Yerbanga R.S., Lefevre T., Cohuet A., Rowley M.J., Ouedraogo J.B., Corces V.G. (2017). Epigenetic regulation of Plasmodium falciparum clonally variant gene expression during development in Anopheles gambiae. Sci. Rep..

[121] Frevert U. (2004). Sneaking in through the back entrance: The biology of malaria liver stages. Trends Parasitol..

[122] Silvie O., Goetz K., Matuschewski K. (2008). A sporozoite asparagine-rich protein controls initiation of Plasmodium liver stage development. PLoS Pathog..

[123] Aly A.S., Lindner S.E., MacKellar D.C., Peng X., Kappe S.H. (2011). SAP1 is a critical post-transcriptional regulator of infectivity in malaria parasite sporozoite stages. Mol. Microbiol..

[124] Aly A.S., Mikolajczak S.A., Rivera H.S., Camargo N., Jacobs-Lorena V., Labaied M., Coppens I., Kappe S.H. (2008). Targeted deletion of SAP1 abolishes the expression of infectivity factors necessary for successful malaria parasite liver infection. Mol. Microbiol..

[125] Helm S., Lehmann C., Nagel A., Stanway R.R., Horstmann S., Llinas M., Heussler V.T. (2010). Identification and characterization of a liver stage-specific promoter region of the malaria parasite Plasmodium. PLoS ONE.

[126] Cogswell F.B. (1992). The hypnozoite and relapse in primate malaria. Clin. Microbiol. Rev..

[127] Voorberg-van der Wel A., Roma G., Gupta D.K., Schuierer S., Nigsch F., Carbone W., Zeeman A.M., Lee B.H., Hofman S.O., Faber B.W. (2017). A comparative transcriptomic analysis of replicating and dormant liver stages of the relapsing malaria parasite Plasmodium cynomolgi. eLife.

[128] Gural N., Mancio-Silva L., Miller A.B., Galstian A., Butty V.L., Levine S.S., Patrapuvich R., Desai S.P., Mikolajczak S.A., Kappe S.H.I. (2018). In Vitro Culture, Drug Sensitivity, and Transcriptome of Plasmodium Vivax Hypnozoites. Cell Host Microbe.

[129] White M.W., Radke J.R., Radke J.B. (2014). Toxoplasma development—Turn the switch on or off?. Cell. Microbiol..

[130] Szatanek T., Anderson-White B.R., Faugno-Fusci D.M., White M., Saeij J.P.J., Gubbels M.-J. (2012). Cactin is essential for G1 progression in Toxoplasma gondii. Mol. Microbiol..

[131] Walker R., Gissot M., Croken M.M., Huot L., Hot D., Kim K., Tomavo S. (2013). The Toxoplasma nuclear factor TgAP2XI-4 controls bradyzoite gene expression and cyst formation. Mol. Microbiol..

[132] Wang J., Dixon S.E., Ting L.-M., Liu T.-K., Jeffers V., Croken M.M., Calloway M., Cannella D., Hakimi M.A., Kim K. (2014). Lysine acetyltransferase GCN5b interacts with AP2 factors and is required for Toxoplasma gondii proliferation. PLoS Pathog..

[133] Hong D.-P., Radke J.B., White M.W. (2017). Opposing Transcriptional Mechanisms Regulate Toxoplasma Development. mSphere.

[134] Radke J.B., Lucas O., De Silva E.K., Ma Y., Sullivan W.J., Weiss L.M., Llinas M., White M.W. (2013). ApiAP2 transcription factor restricts development of the Toxoplasma tissue cyst. Proc. Natl. Acad. Sci. USA.

[135] Croken M.M., Ma Y., Markillie L.M., Taylor R.C., Orr G., Weiss L.M., Kim K. (2014). Distinct strains of Toxoplasma gondii feature divergent transcriptomes regardless of developmental stage. PLoS ONE.

[136] Radke J.B., Worth D., Hong D., Huang S., Sullivan W.J., Wilson E.H., White M.W. (2018). Transcriptional repression by ApiAP2 factors is central to chronic toxoplasmosis. PLoS Pathog..

[137] Huang S., Holmes M.J., Radke J.B., Hong D.-P., Liu T.-K., White M.W., Sullivan W.J. (2017). Toxoplasma gondii AP2IX-4 Regulates Gene Expression during Bradyzoite Development. mSphere.

[138] Klug D., Kehrer J., Frischknecht F., Singer M. (2018). A synthetic promoter for multi-stage expression to probe complementary functions of *Plasmodium* adhesins. J. Cell Sci..

[139] Pino P., Sebastian S., Kim E.A., Bush E., Brochet M., Volkmann K., Kozlowski E., Llinás M., Billker O., Soldati-Favre D. (2012). A tetracycline-repressible transactivator system to study essential genes in malaria parasites. Cell Host Microbe.

[140] Lai B.-S., Witola W.H., El Bissati K., Zhou Y., Mui E., Fomovska A., McLeod R. (2012). Molecular target validation, antimicrobial delivery, and potential treatment of Toxoplasma gondii infections. Proc. Natl. Acad. Sci. USA.

